# Safety and Efficacy of Very Early Conversion to Belatacept in Pediatric Kidney Transplantation with Transplant-Associated Thrombotic Microangiopathy: Case Study and Review of Literature

**DOI:** 10.3390/clinpract14030069

**Published:** 2024-05-16

**Authors:** Ratna Acharya, William Clapp, Kiran Upadhyay

**Affiliations:** 1 Department of Pediatrics, Nemours Children’s Hospital, Orlando, FL 32827, USA; 2 Division of Anatomic Pathology, Department of Pathology, University of Florida, Gainesville, FL 32610, USA; 3 Division of Pediatric Nephrology, Department of Pediatrics, University of Florida, Gainesville, FL 32610, USA

**Keywords:** belatacept, thrombotic microangiopathy, kidney transplantation, eculizumab

## Abstract

The inhibition of co-stimulation during T-cell activation has been shown to provide effective immunosuppression in kidney transplantation (KT). Hence, the conversion from calcineurin inhibitor (CNI) to belatacept is emerging as a potential alternate maintenance immunosuppressive therapy in those with transplant-associated thrombotic microangiopathy (TA-TMA) or in the prevention of TA-TMA. We present a 17-year-old male who presented with biopsy-proven CNI-associated TA-TMA immediately post-KT. The administration of eculizumab led to the reversal of TMA. Tacrolimus was converted to belatacept with excellent efficacy and safety during a short-term follow-up of one year. Further larger controlled studies are required to demonstrate the efficacy of this approach in children who present with early-onset TMA post-KT.

## 1. Introduction

The incidence rate of de novo transplant-associated thrombotic microangiopathy (TA-TMA) in association with calcineurin inhibitor (CNI) is 0.8–3.3%, with most cases being described after kidney transplantation (KT) [1,2,3]. Although TA-TMA occurs most commonly in the initial 3–6 months post-KT, a very early onset of TA-TMA has also been described after KT [4,5]. The treatment consists of a reduction in or discontinuation of CNI, plasmapheresis and the usage of complement inhibitors such as eculizumab [6,7]. Given that the risk of rejection is higher with the CNI discontinuation strategy, many centers add non-CNI-based immunosuppression (IS) in these selected patients who develop CNI-associated TMA [8]. This approach, however, may not provide optimal IS, especially in the immediate post-KT period [8]. Hence, a cytotoxic T-lymphocyte-associated antigen immunoglobulin (CTLA4-Ig) such as belatacept has been studied as a potential alternative to CNI as a maintenance therapy in this subset of patients [9].

CTLA4-Ig blocks the co-stimulation between CD80/CD86 ligands on antigen-presenting cells and CD28 on T cells. Belatacept has been used instead of CNI for maintenance IS in adult KT recipients [9,10]. A Cochrane database review of 1535 adult KT recipients who either received CNI or belatacept as maintenance IS reported no significant difference in the incidences of rejection and graft/patient survival for up to three years following KT [11]. Although the data on the usage of belatacept in pediatric KT recipients are scarce, available studies have shown a good efficacy and safety of both early and late conversion to belatacept [12,13,14].

Here, we report one-year efficacy and safety data of very early conversion from CNI to belatacept in an adolescent KT recipient who developed de novo thrombotic microangiopathy immediately after the initiation of CNI.

## 2. Case Presentation

A 17-year-old adolescent had end-stage renal disease secondary to prune belly syndrome. He had bilateral native kidneys in situ and produced about two liters of urine daily. His bladder regimen consisted of urethral clean intermittent catheterization (CIC) four times daily along with nighttime indwelling catheterization. He underwent an uneventful pre-emptive ABO-compatible deceased donor KT from a standard criteria donor without native nephrectomy. The crossmatch was negative. The warm ischemia time was 25 min and cold ischemia time was 10 h. The panel reactive antibody was 2% prior to the transplant. Both donor and recipients were seropositive for Epstein–Barr virus (EBV). Induction was conducted with a total of 4.5 mg/kg of Thymoglobulin^R^ over three days and 10 mg/kg of pulse steroid. Mycophenolate 600 mg/m^2^/dose twice-daily was started on the day of transplant, and tacrolimus 3 mg twice-daily was started on the first postoperative day (POD) after the serum tacrolimus decreased to 50% of the pre-transplant value, as per the transplant center’s protocol.

A follow-up renal function test showed a gradual rise of serum creatinine 12 h following the initiation of tacrolimus on the first POD. Blood urea nitrogen and serum creatinine peaked at 126 mg/dL and 9.5 mg/dL, respectively, on the fifth POD with hyperkalemia and acidosis. The serum tacrolimus trough levels ranged from 3 to 4 ng/mL during that time, despite being on tacrolimus up to 6 mg twice daily on POD 5. Urine output remained at the pre-transplant level. The blood count showed progressive thrombocytopenia which was initially attributed to Thymoglobulin. However, the patient also had elevated serum lactate dehydrogenase (LDH) and undetected haptoglobin. The peripheral smear showed schistocytes. Other laboratory investigations are shown in Table 1. The simultaneous testing of pre- and post-transplant sera showed no post-transplant donor-specific antibodies (FlowPRA beads). An atypical hemolytic uremic syndrome (HUS) panel was sent.

The initial renal allograft sonogram on the day of transplant showed good blood flow and no evidence of hydronephrosis. However, on the first POD, the doppler sonogram was concerning for elevated resistive indices at all three poles of the transplant. A mercaptoacetyltriglycine renal scan on the third POD showed a good flow of the dye to the transplant kidney but with progressive accumulation, consistent with acute tubular necrosis.

**Figure 1:**
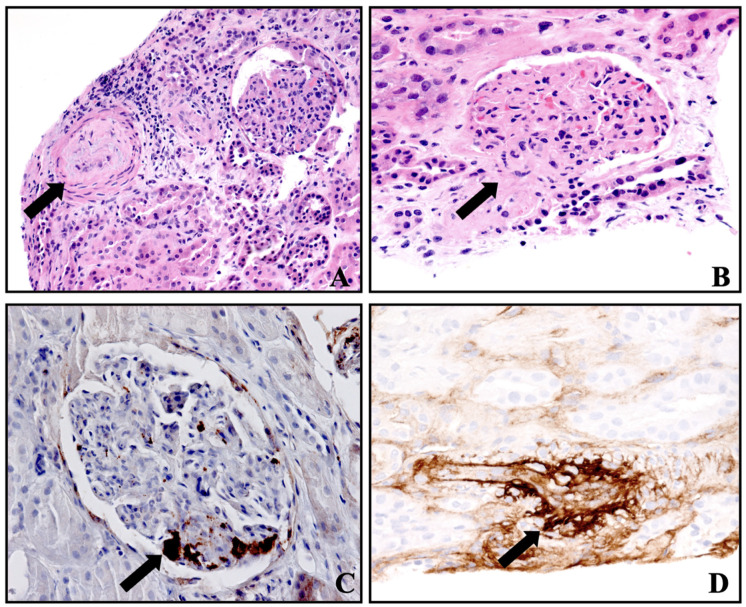
Histology of transplant kidney showing changes in thrombotic microangiopathy. (**A**) Small artery with edematous intimal thickening and severe luminal narrowing (light microscopy, H&E, 200×), (**B**) glomerular capillary closure by swollen endothelial cells and thrombotic occlusion of hilar arteriole (light microscopy, H&E, 400×), (**C**) glomerular platelet microthrombi (immunohistochemistry, CD61 staining, 400×), (**D**) small artery thrombus with staining for factor VIII (immunohistochemistry, 400×).

A transplant kidney biopsy performed on the fourth POD showed 16 glomeruli with glomerular platelet microthrombi, glomerular capillary closure by swollen endothelial cells and the thrombotic occlusion of the hilar arteriole (Figure 1B,C), focal acute tubular injury characterized by tubular dilatation and epithelial simplification and casts, without frank tubular necrosis. Two small arteries showed edematous intimal thickening and severe luminal narrowing with fibro-intimal proliferation without definite vascular thrombi (Figure 1A). The increased expression of factor VIII was noted in the small arteries and arterioles by immunohistochemistry without any vasculitis (Figure 1D). The immunostain for BK virus was negative. There was no peritubular capillary C4d deposition. There were no definite areas of segmental sclerosis, necrosis or crescent formation. The interstitial fibrosis/tubular atrophy was 15%. There were scattered mononuclear cell infiltrates, consisting of both CD3 and CD68 immunoreactive T cells and macrophages, respectively. Electron microscopy showed glomerular endothelial cytoplasmic swelling with several glomerular loops containing intraluminal mononuclear cells. There were no electron-dense deposits. The glomerular basement membrane showed no splitting or multilaminations. Segmental podocyte foot process effacement was seen. These vascular and glomerular findings were suggestive of thrombotic microangiopathy along with acute tubular injury.

**Figure 2:**
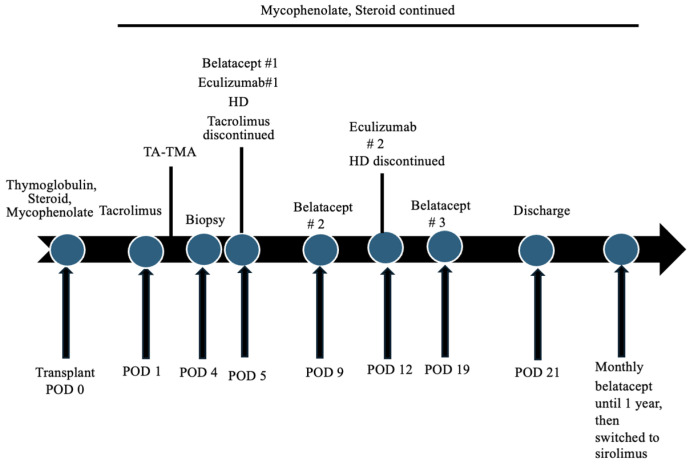
Timeline of events following kidney transplantation. POD: postoperative day; TA-TMA: transplant-associated thrombotic microangiopathy; HD: hemodialysis.

He also developed transient stage II hypertension immediately after transplantation which was attributed to fluid overload, pain, the usage of CNI and steroids and possibly the development of TMA. Hypertension was well controlled with amlodipine and labetalol. Hemodialysis (HD) was started on the fifth POD for severe uremia. He received a total of five sessions of HD. Due to the likelihood of post-transplant CNI-associated TMA and the concerns with the inferior therapeutic efficacy of other oral non-CNI-based immunosuppression, tacrolimus was switched to intravenous (IV) belatacept 10 mg/kg after receiving four dosages of tacrolimus. His Epstein–Barr virus (EBV) capsid antigen IgG was positive. The subsequent dosages of IV belatacept 10 mg/kg were administered four days later and then at the end of the second week after the first dose. He also received two dosages of IV eculizumab 600 mg, a week apart, after the administration of penicillin and the meningococcal vaccine. Figure 2 shows the timeline of events following the transplantation. The platelet count, haptoglobin and LDH normalized within a week after the administration of the first dose of eculizumab. Plasmapheresis was not performed.

The renal function gradually improved, and he was discharged home in a stable condition three weeks after the transplant. Serum creatinine at discharge was 2.8 mg/dL, and the complete blood count showed normal blood counts. LDH was 247 IU/L, and haptoglobin normalized (Table 1). The hemodialysis catheter was removed. Urethral CIC was continued every four hours along with indwelling overnight catheterization. He was continued on a monthly 5 mg/kg of IV belatacept infusions along with 300 mg/m^2^/dose twice-daily mycophenolate and 10 mg prednisone daily. Anti-infective prophylaxis consisted of nystatin, valganciclovir and trimethoprim–sulfamethoxazole. The donor-derived cell-free DNA (dd-cf DNA) a month post-KT was 0.87% (normal < 0.70%, Eurofins Transplant Genomics, Framingham, MA, USA).

A follow-up of the atypical HUS panel showed no autoantibodies to factor H and I, normal serum factor H and I levels and an elevated soluble complement 5B-9 (SC5B-9) level (Table 1). The atypical HUS genetic susceptibility panel showed the absence of the deletion/duplication of *C3*, *C4BPA*, *CD46*, *CD59*, *CFB*, *CFH*, *CFI*, *CFHR5*, *DGKE*, *THBD*, *PLG* and *MMACHC* genes (Cincinnati Children’s Clinical Laboratories, Cincinnati, OH, USA). Over the course of one year, the BK, cytomegalovirus and EBV polymerase chain reactions remained negative. Also, the donor-specific antibodies (DSAs) and proteinuria were closely monitored, which were negative. Allograft function remained stable with baseline serum creatinine around 1.2–1.4 mg/dL. At one year post-KT, the estimated glomerular filtration rate (eGFR) was 76 mL/min/1.73 m^2^. Blood pressures remained stable on labetalol monotherapy.

**Figure 3:**
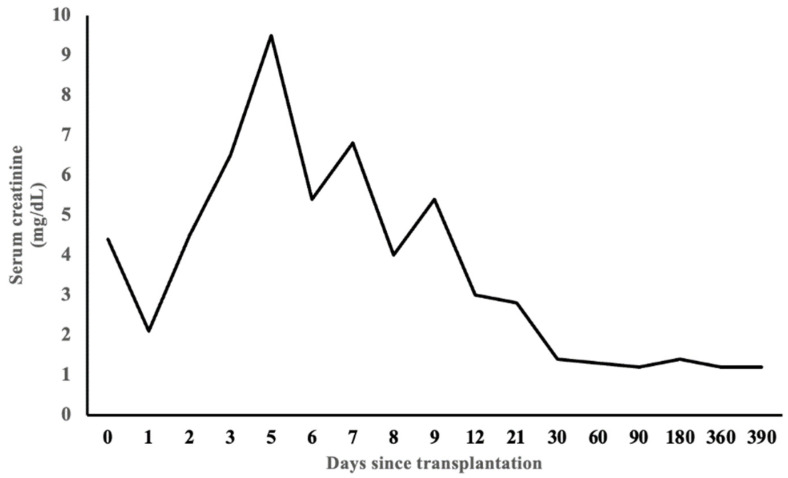
Changes in serum creatinine after kidney transplantation.

After one year of transplant, monthly belatacept was switched to oral sirolimus upon the family’s request given that the family lived 300 miles away from the center. Also, the treatment team was confident with his oral medication compliance. Allograft function during the one-month follow-up after switching to sirolimus showed stable baseline serum creatinine (Figure 3).

## 3. Discussion

The current standard IS regimen in KT recipients at most centers includes CNI and antiproliferative agents with or without steroids [15]. However, the longer-term adverse effects of CNI include hypertension, dyslipidemia, diabetes, nephrotoxicity and drug-induced TMA, among others [16]. Hence, an alternate agent without these side effects would be beneficial. Belatacept-treated KT recipients were more likely to have better graft function and less likely to have renal scarring, hypertension, dyslipidemia and diabetes [11].

In two phase 3 studies (Belatacept Evaluation of Nephroprotection and Efficacy as First-line Immunosuppression Trial [BENEFIT] and BENEFIT–extended criteria donors [BENEFIT-EXT]), the adult KT patients who received de novo belatacept had comparable patient/graft survival and superior renal function as compared to cyclosporine-treated patients at 12 and 36 months post-KT [17,18,19,20]. Also, longer follow-up data for up to 7 years from the BENEFIT and BENEFIT-EXT have shown a sustained efficacy and safety of belatacept [21].

Both the early and late conversion of CNI to belatacept have been shown to be efficacious and safe in non-highly sensitized adult KT recipients [22,23]. Ashman et al. reported a case of biopsy-proven post-transplant de novo TMA in association with cyclosporin, tacrolimus and sirolimus in an adult KT recipient [2]. Belatacept was used as a rescue therapy on day 84 post-KT followed by monthly infusions. During a nine-month follow-up after KT, renal function remained stable with no evidence of TMA [2]. Among highly sensitized patients, the rejection-free survival after conversion may be lower in the first year post-KT. However, this observation seems to be non-persistent over a five-year period [24]. Also, the early rejection episodes seen with belatacept in some studies have been shown to be mitigated with alemtuzumab induction and sirolimus maintenance therapy [17,25]. Our immunologically low-risk patient received Thymoglobulin^R^ induction and had no rejection episodes after conversion from CNI to belatacept.

Limited data exist on the usage of belatacept in children. Moudgil et al. performed a phase I trial of a single dose of belatacept to assess its pharmacokinetics (PK) and pharmacodynamics (PD) in nine 12–17-year-old KT recipients who were receiving CNI-based IS. The study demonstrated similar PK and PD as compared to the healthy adult volunteers and adult KT recipients receiving single and multiple doses of belatacept, respectively [26]. Lerch et al. reported six children with belatacept conversion (median conversion time 7.5 months post-KT) with an increase in the estimated glomerular filtration rate (eGFR) among those who were switched within the first three months; others had a stable eGFR [14]. Similarly, Blew et al. reported the efficacy of belatacept in three adolescent KT recipients who received alemtuzumab induction, with good clinical outcomes for up to 20 months post-KT [12]. Garro et al. reported that the conversion to belatacept (median 27.5 months post-KT) permitted CNI minimization or elimination and a stable or improved eGFR, without graft rejection/loss in ten pediatric KT recipients [13]. BK viremia, transient cytomegalovirus viremia and variable DSA occurred in their cohort. In our patient, no infections or rejections occurred. Being seropositive for EBV posed a low risk for central nervous system (CNS) post-transplant lymphoproliferative disorder (PTLD), as there is a potential for CNS PTLD with the usage of belatacept in patients who are seronegative for EBV.

The most common causes of de novo TMA following KT are malignant hypertension, calcineurin inhibitors, infections and malignancies among others [27,28]. Thrombocytopenia, acute kidney injury and microangiopathic hemolytic anemia are the classic presenting features but may not be present in all cases [29]. A gradually worsening renal insufficiency and hypertension may be the only manifestations in some patients [30]. The graft loss rate can be as high as 40% at two years [31]. Patient survival may also be poor with a three-year survival rate of 50% (13). The discontinuation of CNI therapy is the main strategy. Also, various studies have described the role of eculizumab in CNI-associated post-KT TMA [6,32]. Along with belatacept, Merola et al. reported the efficacy of five dosages of eculizumab in a highly sensitized adult patient who developed CNI-induced TMA within a week after KT [5]. The patient had stable renal function two years after KT on monthly belatacept infusions along with mycophenolate and steroids. Our report is unique in that we reported the one-year efficacy and safety of the very early conversion of belatacept in a pediatric KT recipient with de novo tacrolimus-associated TMA. Also, our patient received only two weekly doses of eculizumab unlike the prior reported study. The utility of eculizumab may be more pronounced in those with TMA as a manifestation of antibody-mediated rejection (ABMR), more so in ABO-incompatible transplantation [33,34,35]. The patient described in this report underwent ABO-compatible transplantation, did not develop DSA and the transplant kidney biopsy showed no evidence of ABMR. With regards to the subset of patients without concomitant ABMR, larger studies are needed to determine the efficacy of anti-complement therapy.

The exact pathogenesis of CNI-associated TMA is unclear. The direct toxicity of the CNI causing vasoconstriction leading to renal ischemia and endothelial injury is the most widely accepted hypothesis and can occur hours after exposure [36,37,38]. The histological features are microthrombi in the glomeruli and arterioles, fibrin in the glomeruli, the thickening and double contours of the glomerular basement membrane, the fibrinoid necrosis of the glomeruli and arteriolar endothelial swelling [39]. As described in this report, features of concurrent acute tubular injury can be seen [40]. The CNI trough levels have not been shown to be predictive for the development of TMA [30]. An underlying complement dysregulation may or may not be present in these patients [28,41]. Our patient did not have demonstrable genetic complement abnormalities. However, the absence of genetic abnormalities does not exclude the role of complement activation in secondary hemolytic uremic syndrome [42]. Also, the serum complement profile may be normal, and hypocomplementemia may develop with time [38]. One comparative study studied the prevalence of genetic complement abnormalities in those with secondary HUS and in healthy people and concluded that there was no increased risk of complement genetic variants in secondary HUS [28].

### Limitations

We did not see widespread vascular thrombi, but this could have been due to sampling error and perhaps very early sampling after clinical presentation. Other features of TMA were present as described in the biopsy findings. Although post-transplant TMA can be the result of severe hypertension, our patient only had transient mild hypertension and was well controlled with antihypertensives. Also, the rapidity of the onset of the deterioration of renal function after the initiation of tacrolimus led to a higher index of suspicion for CNI-associated TMA. Moreover, TMA resolved promptly with the administration of eculizumab and did not recur with switching from CNI to belatacept. However, we could not definitely establish the cause-and-effect relationship between CNI and TA-TMA. Also, the follow-up was only for about a year. Hence, a longer follow-up would be important to demonstrate the persistent efficacy and safety of belatacept. Also, one could argue that in this patient who received eculizumab, stable allograft function could have been attained even without the usage of belatacept. Although eculizumab may have anti-rejection effects, future studies are needed to support the efficacy of a few doses of eculizumab (two dosages in this case) in maintaining allograft function over a year or so (such as in this report) without other additional immunosuppressive agents such as belatacept, in patients with likely CNI-associated TA-TMA.

## 4. Conclusions

The usage of belatacept in children seems to be a prominent alternate agent to CNI in those who have sustained post-KT CNI-associated TMA. The short-term efficacy and safety of the conversion to belatacept seems to be excellent. However, larger longer-term prospective studies are needed to further support the widespread use of belatacept as an alternate maintenance agent in the pediatric KT population.

## Figures and Tables

**Table 1:** Initial and follow-up investigations showing laboratory and imaging results.

Investigations	Initial Values	Values at Discharge	Normal Values
WBC count	10.2 × 10^9^/L	6.2 × 10^9^/L	4–11 × 10^9^/L
Hemoglobin	7.3 g/dL	12.1 g/dL	10.5–13.5 g/dL
Platelet count	35 × 10^9^/L	310 × 10^9^/L	150–450 × 10^9^/L
LDH	1452 IU/L	247 IU/L	135–225 IU/L
Haptoglobin	<30 mg/dL	110 mg/dL	40–215 mg/dL
Peripheral smear	Few schistocytes		
Direct antiglobulin test, C3 and IgG	Negative		
ADAMTS13 activity	80%		>60%
C3 complement	104 mg/dL		87–200 mg/dL
C4 complement	18 mg/dL		13–50 mg/dL
SC5B-9 level	480 ng/mL		≤244 ng/mL
Genetic testing for atypical HUS	Negative for complement mutations or deficiencies		
Serum sodium	141 mmol/L	140 mmol/L	135–145 mmol/L
Serum potassium	4.3 mmol/L	4.2 mmol/L	3.5–4.5 mmol/L
Serum bicarbonate	23 mmol/L	22 mmol/L	22–30 mmol/L
BUN	126 mg/dL	52 mg/dL	6–21 mg/dL
Serum creatinine	9.5 mg/dL	2.8 mg/dL	0.20–0.43 mg/dL
Serum calcium	8.3 mg/dL	10.4 mg/dL	8.4–10.2 mg/dL
Serum phosphorus	5.8 mg/dL	4.4 mg/dL	4.3–6.8 mg/dL
Serum albumin	3 g/dL	4.1 g/dL	3.5–5.2 g/dL
EBV DNA PCR, CMV DNA PCR, BK virus DNA PCR	Negative		
Urinalysis	2 RBC/hpf, 2 WBC/hpf, trace proteinuria, negative nitrites and leukocytes, pH 7, specific gravity 1.025		
Renal transplant sonogram	11.6 cm in length with no hydronephrosis, minimal perinephric fluid collection, normal corticomedullary differentiation and normal echogenicity Resistive indices: ⇒ Main renal artery: 0.87 ⇒ Superior pole arcuate artery: 0.81 ⇒ Interpole arcuate artery: 0.83 ⇒ Inferior pole arcuate artery: 0.79		

WBC: White blood cell; LDH: lactate dehydrogenase; ADAMTS13: a disintegrin-like metalloproteinase with thrombospondin motif type 1; SC5B-9: soluble complement 5b-9; HUS; hemolytic uremic syndrome; BUN: blood urea nitrogen; EBV: Epstein–Barr virus; CMV: cytomegalovirus; PCR: polymerase chain reaction.

## Data Availability

Data are available.
